# *In Utero* Bisphenol A Exposure Induces Abnormal Neuronal Migration in the Cerebral Cortex of Mice

**DOI:** 10.3389/fendo.2016.00007

**Published:** 2016-02-01

**Authors:** Wenting Ling, Toshihiro Endo, Ken-ichiro Kubo, Kazunori Nakajima, Masaki Kakeyama, Chiharu Tohyama

**Affiliations:** ^1^Laboratory of Environmental Health Sciences, Center for Disease Biology and Integrative Medicine, Graduate School of Medicine, The University of Tokyo, Tokyo, Japan; ^2^Department of Anatomy, Keio University School of Medicine, Tokyo, Japan; ^3^Laboratory for Systems Neuroscience and Preventive Medicine, Faculty of Human Sciences, Waseda University, Tokorozawa, Japan; ^4^Environmental Biology Laboratory, Faculty of Medicine, University of Tsukuba, Tsukuba, Japan

**Keywords:** bisphenol A, low dose, brain development, cerebral cortex, environmental chemicals, neuronal migration

## Abstract

Bisphenol A (BPA) has been known to have endocrine-disrupting activity to induce reproductive and behavioral abnormalities in offspring of laboratory animal species. However, morphological basis of this abnormality during brain development is largely unknown. Cerebral cortex plays a crucial role in higher brain function, and its precisely laminated structure is formed by neuronal migration. In the present study, transfecting a plasmid (pCAG-mCherry) by *in utero* electroporation (IUE), we visualized developing neurons and investigated the possible effects of *in utero* BPA exposure on neuronal migration. Pregnant mice were exposed to BPA by osmotic pump at estimated daily doses of 0, 40 (BPA-40), or 400 (BPA-400) μg/kg from embryonic day 14.5 (E14.5) to E18.5. IUE was performed at E14.5 and neuronal migration was analyzed at E18.5. Compared with the control group, neuronal migration in the cortical plate was significantly decreased in the BPA-40 group; however, there was no significant difference in the BPA-400 group. Among several neuronal migration-related genes and cortical layer-specific genes, TrkB in the BPA-400 group was found significantly upregulated. In conclusion, *in utero* exposure to low BPA dose was found to disrupt neuronal migration in the cerebral cortex in a dose-specific manner.

## Introduction

Bisphenol A (BPA, 4,4′-dihydroxy-2,2-diphenylpropane) is a monomer used worldwide for manufacturing plastics, such as polycarbonates and epoxy resins. Humans are widely exposed to BPA *via* leaching from plastic bottles, sealants for canned food, and other environmental sources. Low doses of BPA exposure during the perinatal period can result in numerous effects on health, ranging from reversible physiological responses to more long-term adverse effects. Exposure to BPA early in life has been reported to be associated with behavioral problems in children (1–3), presumably due to their limited capacity to metabolize BPA and to the fact that the blood–brain barrier is not fully developed. BPA has also been suggested to have adverse effects on neuronal development in human infants (4). Furthermore, animal studies have shown that offspring born to dams exposed to low doses of BPA during gestation and the early postnatal period had abnormal brain morphologies (5–9). However, the mechanisms by which maternal BPA exposure affects embryonic brain development are still largely unknown.

The laminated structure of the cerebral cortex is formed by highly tuned neuronal migration. Perturbations of this neuronal migration result in neurological and developmental abnormalities. Although neuronal migration has been suggested as a target of chemical exposure, it has not been widely studied in the context of developmental neurotoxicity. A few studies have reported that neuronal migration can be interrupted by environmental chemicals, such as methylmercury (10, 11) and toluene (12). The aim of the present study was to examine the possible effects of prenatal exposure to low doses of BPA on the process of neuronal migration. For this, we used *in utero* electroporation (IUE), a gene-transfer technique that enabled us to introduce fluorescent protein expression vectors into neuronal progenitor cells and visualize the process of migration (13, 14). We found that prenatal exposure to BPA interrupted neuronal migration in the cerebral cortex in a dose-specific manner.

## Materials and Methods

Pregnant ICR mice were purchased from CLEA Japan (Tokyo, Japan). The day of vaginal plug observation was designated as E0.5. Mice were housed in an animal room maintained at a temperature of 22–24°C, humidity at 40−60%, and under 12-h light/12-h dark cycles (lights on and off at 0800 and 2000 hours, respectively). Food (Labo MR Stock, Nosan, Yokohama, Japan) and water were provided *ad libitum*. Pregnant mice were exposed to BPA (Wako Pure Chemical Ind., Osaka, Japan) at a daily dose equivalent to 0, 40, or 400 μg/kg b.w. from E14.5 to E18.5 by implanting an osmotic pump (Alzet, Micro-Osmotic Pump, Model 1007D, Cupertino, CA, USA) into the peritoneal cavity. According to the doses given to the dams, the control and BPA exposed groups were named as Control, BPA-40, and BPA-400, respectively. The experimental protocols for the animal experiments were approved by the Animal Care and Use Committee of the University of Tokyo.

A plasmid (pCAG-mCherry, a kind gift from Dr. Masanori Matsuzaki at the National Institute for Basic Biology, Okazaki, Japan) was purified using the EndoFree Plasmid Kit (Qiagen K.K., Tokyo, Japan) according to the manufacturer’s protocol. The purified plasmid was diluted with phosphate-buffered saline (PBS) to a final concentration of 3 μg/μl before use.

*In utero* electroporation was performed at E14.5, as described previously (13, 14). Briefly, time-pregnant mice were deeply anesthetized by an intraperitoneal injection of sodium pentobarbital solution (Dainippon Sumitomo Pharma, Osaka, Japan) at a dose of 50 mg/kg b.w. After the uterus was carefully pulled out from the abdominal cavity, an aliquot (approximately 1 μl) of plasmid solution colored by 0.01% fast green was injected into the lateral ventricle of the embryo and was transfected by electroporation (30−35 V, 50 ms, four pulses) using a square wave electroporator (CUY21SC, Nepa Gene Co., Chiba, Japan) with a forceps-type electrode (CUY650P5). The uterus was returned to the abdominal cavity, and an osmotic pump was implanted in the peritoneal cavity, followed by a closure of the abdomen with sutures. In this study, plasmids were successfully transfected into more than half of embryonic brains.

Mice were sacrificed at E18.5. Embryonic brains were collected, fixed with 4% paraformaldehyde (PFA) in PBS overnight at 4°C, and immersed consecutively in 20 and 30% sucrose in PBS at 4°C. Then, brains were embedded in O.C.T. compound (Sakura Finetek, Tokyo, Japan), and stored at −80°C until analysis. Frozen brains were cut into 20 μm thick coronal sections by cryostat (CM3050S, Leica Microsystems K.K., Tokyo, Japan).

To visualize the cortical plate (CP) boundary, microtubule-associated protein 2 (Map2) immunostaining was performed. Briefly, brain tissue sections were washed in PBS containing 0.05% Triton X-100 (PBST) and fixed in 4% PFA for 10 min. After blocking with 3% bovine serum albumins in PBST at room temperature for 1 h, the brain sections were incubated with anti-MAP2 antibodies that were conjugated to Alexa Fluor 488 (Merck Millipore Japan Headquarters, Tokyo, Japan) for 3 h. Following additional washing with PBST, the sections were mounted with 4′,6-diamidino-2-phenylindole (DAPI, Vector Laboratories, Burlingame, CA, USA). Images were acquired using a Leica microscope (DM6000 B, Leica Microsystems K.K.), processed with Neurolucida (MBF Bioscience) and Image-J (NIH) software.

Cell distribution in the CP was evaluated using a bin analysis, as described previously (15). The CP was equally divided into 10 bins in which the bin closest to the ventricle was numbered as Bin 1 and the bin closest to the pia mater was numbered as Bin 10. In each bin, the number of fluorescent cells (mCherry-positive cells) was estimated as a percentage of the total number in all 10 bins, using Image-J software (National Institute of Health, Bethesda, MD, USA). Embryonic brains that were successfully transfected with fluorescent protein vectors were selected for cell migration analysis. One to three brains from each litter were randomly selected, and total two to four litters in each dosed group were analyzed.

For body weight and mRNA analyses, another set of BPA-exposed pregnant mice was used. At PND 0, we checked the litter size and body weight and randomly selected one male from each litter, followed by the analysis of a total of six pups in each dosed group. Forebrains were dissected, snap frozen in liquid nitrogen, and stored at −80°C until analysis.

Total RNAs were extracted using the RNeasy Mini Kit (Qiagen) according to the manufacturer’s instructions. cDNA synthesis was performed using the PrimeScript RT reagent Kit (Takara, Otsu, Japan). Quantitative real-time PCR was performed using the Thunderbird qPCR mix (Toyobo, Osaka, Japan) and LightCycler (Roche Diagnostic Co., Tokyo, Japan). Primer design and specificity check were performed by Primer-BLAST (NCBI, Bethesda, MD, USA). The mRNA expression of the target gene in each sample was normalized with glyceraldehyde-3-phosphate dehydrogenase (GAPDH).

For statistical analysis, one-way ANOVA (for litter size, body weight, and mRNA expression) and two-way ANOVA (for neuronal migration) followed by the Tukey–Kramer’s *post hoc* test were used. All data are expressed as mean ± SEM. *p*-Values <0.05 were considered statistically significant.

## Results

There was no significant difference in litter size between control dams and BPA-exposed dams. In addition, no statistical differences in body weight were observed between groups for either sex at PND 0 (Table 1).

**Table 1:** **Litter size and pup body weight after *in utero* BPA exposure^a^**.

Group^b^	Litter size			Body weight (g)	
	All pups	Male	Female	Male	Female
Control	14.8 ± 0.8	6.67 ± 0.61	8.17 ± 0.79	1.67 ± 0.05	1.60 ± 0.04
BPA-40	13.7 ± 1.5	5.83 ± 1.10	7.83 ± 1.01	1.81 ± 0.05	1.71 ± 0.05
BPA-400	15.0 ± 0.7	8.50 ± 0.80	6.50 ± 1.09	1.77 ± 0.03	1.69 ± 0.03

*^a^Data are shown as mean ± SEM*.

*^b^*n* = 6 dams per treatment*.

**Figure 1:**
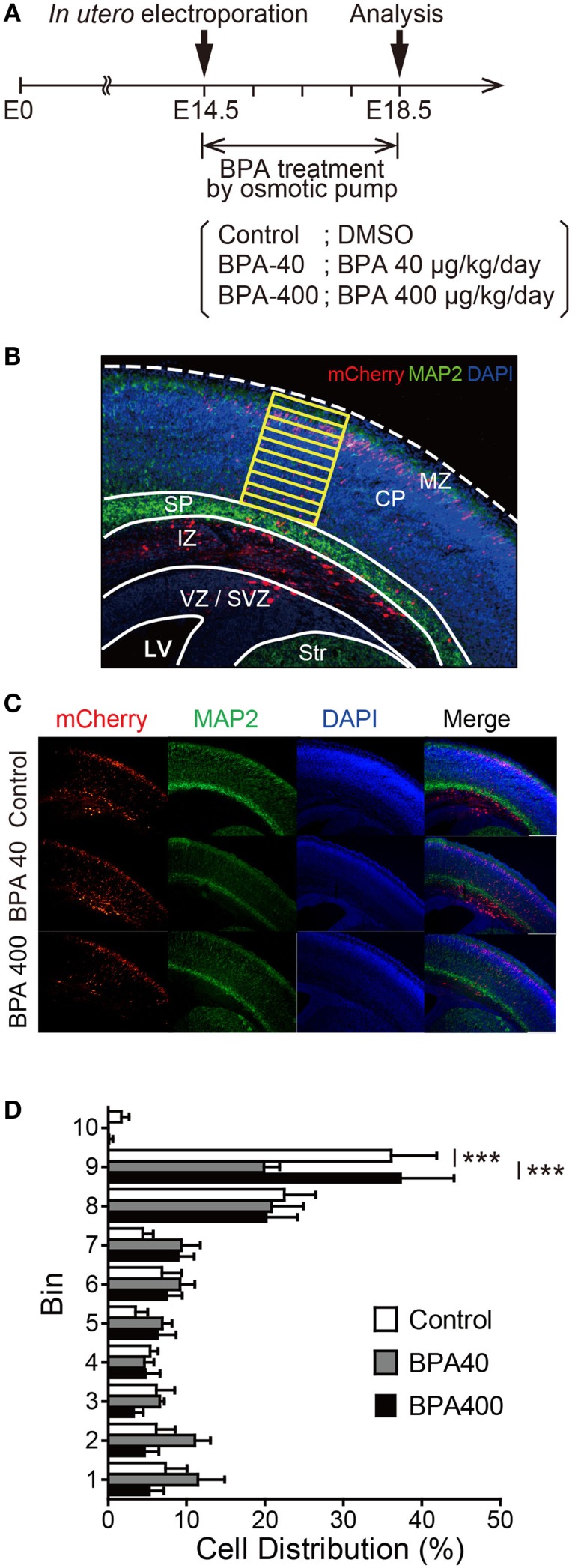
**Effects of prenatal BPA exposure on neuronal migration in the developing cerebral cortex**. **(A)** Diagram of the experiment design. **(B)** Bin analysis was performed to evaluate differences in cell distribution. MZ, marginal zone; CP, cortical plate; SP, subplate; IZ, intermediate zone; SVZ, subventricular zone; VZ, ventricular zone; LV, lateral ventricle; Str, striatum. **(C)** Representative photographs of brain sections in the Control, BPA-40, and BPA-400 groups. **(D)** Distribution of mCherry-positive neurons in the CP at E18.5. Data are shown as mean ± SEM, *n* = 4 (control), *n* = 5 (BPA-40), *n* = 5 (BPA-400), ***p < 0.01.

To investigate whether *in utero* BPA exposure affects neuronal migration, IUE was performed at E14.5 to introduce a fluorescent protein expression vector (pCAG-mCherry) into neural progenitor cells, and the distribution of mCherry-positive neurons was analyzed at E18.5 in the three groups (Control, BPA-40, and BPA-400) (Figure 1A). At E18.5, mCherry-positive cells were found in ventricular zone (VZ), subventricular zone (SVZ), intermediate zone (IZ), and CP (Figures 1B,C). Beneath the subplate (SP), not only mCherry-positive cells but also axons of projection neurons overlapped together exhibiting intense mCherry fluorescent signals, and it was very difficult to differentiate each single mCherry-positive cell from mCherry signals in IZ, so only the mCherry-positive cells migrated into the CP were subjected to a bin analysis to examine the cell distribution in CP (Figure 1B). In the control group, the majority of mCherry-positive neurons were located in layers II/III (corresponding to Bins 8 and 9) of the cerebral cortex. In the BPA-40 group, the percentage of mCherry-positive neurons in Bin 9 was significantly lower than that in the control group or the BPA-400 group. There were no significant differences in the distribution of mCherry-positive neurons in the CP between the BPA-400 group and the control group (Figure 1D). These data show that prenatal exposure to BPA suppresses neuronal migration in a dose-specific manner.

**Figure 2:**
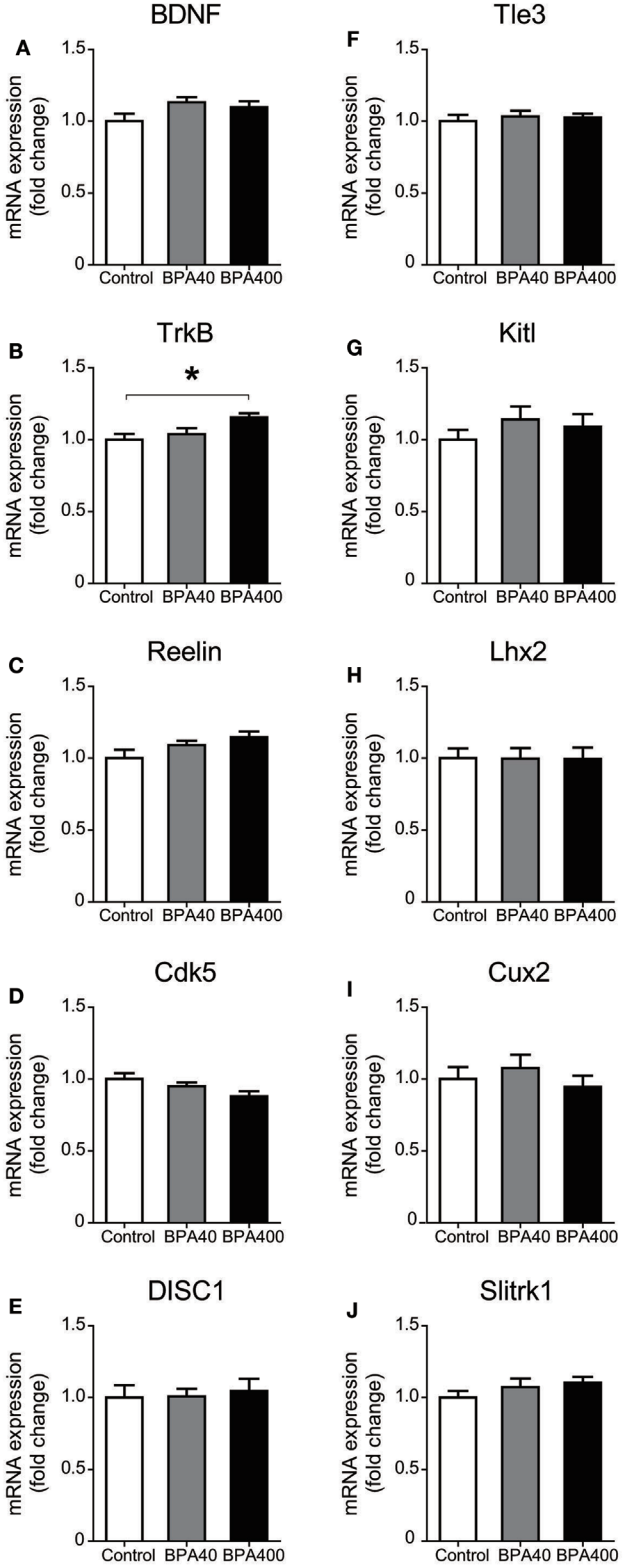
**mRNA expression of neuronal migration-related genes [BDNF (A), TrkB (B), Reelin (C), Cdk5 (D), and DISC1(E)] and cortical layer-specific genes [Tle3 (F), Kitl (G), Lhx2 (H), Cux2 (I), and Slitrk1 (J)] in mouse forebrain**. Data are shown as mean ± SEM, *n* = 6 per treatment, *p < 0.05.

Neuronal migration is guided by various molecular cues. Thus, we examined the forebrain mRNA expression of genes that are known to be important for neuronal migration and layer formation, such as brain-derived neurotrophic factor (BDNF), neurotrophic tyrosine kinase receptor type 2 (TrkB), Reelin, cyclin-dependent kinase 5 (Cdk5), and disrupted in schizophrenia 1 (DISC1), and the neocortical layer II/III laminar-specific genes, such as transducin-like enhancer of split 3, homolog of Drosophila E (Tle3), kit ligand (Kitl), LIM homeobox protein 2 (Lhx2), cut-like homeobox 2 (Cux2), and SLIT and NTRK-like family, member 1 (Slitrk1) (Figure 2). TrkB, a receptor for neurotrophins, was significantly increased in the BPA-400 group compared with the control group. No significant difference in the mRNA expression of other genes was observed among the control, BPA-40, and BPA-400 groups.

## Discussion

In this study, we used IUE to transfect neural progenitor cells with plasmids that express fluorescent protein, and found that *in utero* low doses of BPA exposure significantly perturbed neuronal migration in the embryonic cerebral cortex in mice. The neurons born in VZ at E14.5 are mostly excitatory projection neurons that undergo radial migration in the cerebral cortex before reaching their final destination in layers II/III. Neurons located in layers II/III are commissural projection neurons extending axons to the opposite hemisphere across the corpus callosum. It has been reported that abnormalities in higher brain function observed in various neurological diseases are considered to be associated with the inappropriate positioning of neurons, which in turn leads to inaccurate projections and impairment of synaptogenesis (16, 17). Thus, it is possible that abnormal neuronal migration observed after low doses of BPA exposure could be a cause of abnormal brain function, but the direct link between delayed neuronal migration and high brain functions warrant future studies.

A previous study reported that BPA exposure from E0.5 at a daily dose of 20 μg/kg b.w. significantly decreased the number of 5-bromodeoxyuridine (BrdU)-positive cells in the VZ at E14.5 and E16.5 and increased the number of BrdU-positive cells in the CP at E14.5 compared with the control group, indicating that prenatal BPA exposure accelerates neuronal migration (18). However, the same research group also reported that when BrdU-positive cells were labeled at E14.5 in mice exposed to BPA *in utero*, there was subsequently a significant increase in labeled cells in cortical layers V and VI and a decrease in labeled cells in layer IV when the brains were examined at postnatal week 3 (19). Another *in vitro* study reported that BPA treatment increased the tangential migration of interneurons in cortical slices (20). In cerebral cortex, inhibitory neurons and excitatory neurons are born at different time periods and different places. Inhibitory neurons are born in the ganglionic eminence and migrate tangentially, whereas excitatory neurons are born in the VZ and migrate radially. The differences between results from previous studies and our study may be due to different exposure durations, doses, and neuronal types studied. The present study showed that the prenatal BPA exposure suppressed radial migration of excitatory neurons in the developing cerebral cortex.

The daily doses of 40 and 400 μg/kg used in this study are low doses compared with 5 mg/kg/day, which is regarded as the cutoff dose for low-dose effects regardless of the exposure route and duration (21). The observation of the present study, an abnormal neuronal migration in the mouse embryonic brain, can be added to the list of toxic phenotypes induced by low doses of BPA. Our study provides an example of a dose-specific response to BPA, as shown by the distinct disturbance of neural migration in the BPA-40 group that was absent in the BPA-400 group. A number of previous studies have investigated the effects of low doses of BPA and have shown that the dose–response curve has an inverted U-shape (22, 23). BPA can bind to the estrogen receptor and has been shown to have estrogenic properties. As hormones are known to act in a non-monotonic dose–response manner, the low-dose-specific response to BPA may be regulated by interactions between BPA and hormone receptors (22, 23). For further study, the mode of action of BPA needs to be studied extending a dose range that includes multiple lower BPA doses.

In order to investigate the molecular basis of BPA-induced impairment of neuronal migration observed in the developing brain, we analyzed several migration guidance genes and neuron-specific markers for layer II/III. We identified a significantly enhanced gene expression of TrkB upon high dose BPA exposure. TrkB is a receptor for neurotrophins, which mediate neuronal migration, differentiation, and survival through beneficial trophic effects (24). Therefore, a plausible explanation for the dose-specific effects of BPA on neuronal migration would be that compensatory mechanisms may have been triggered in the BPA-400 group, such that higher TrkB expression minimized the effects of BPA exposure on neuronal migration. However, in order to elucidate mechanisms of BPA-induced abnormal neuronal migration, gene expressions of other neurotrophins (NGF, NT3, and NT4/5) and receptors (Trk A and Trk C) in specific brain regions, such as CP, need to be investigated in future studies. Migration guidance genes, such as Reelin, and several layer II/III-specific genes did not show any significant differences in expression between the control and BPA-40 groups. Because of the limited time point of the determination of mRNA levels on PND 0, it can be speculated that BPA may have disrupted expression of layer-specific genes in later time points. A recent study showed that perinatal exposure to BPA in mice at levels relevant to those exposed to humans transgenerationally altered behaviors and gene expression in brains, including expression of genes for several estrogen receptors, oxytocin, and vasopressin (25). Another recent study (9) showed reduction in overall length and branching number of basal dendrites of hippocampal CA1 pyramidal neurons in 3-week-old mouse pups, and spine densities in aged mice, both of which were born to dams administered BPA (40 or 400 μg/kg per day) during gestation. However, in the present study, we did not find any altered expression in genes, except TrkB as described above, that are relevant to neuronal migration or morphogenesis in BPA-exposed groups. The link of micromorphologically altered neuronal development with the molecular basis warrants prospective studies.

## Author Contributions

WL, TE, MK, and CT conceived this study. WL and TE performed experiments. WL analyzed experimental data. WL, TE, MK, and CT interpreted the data. K-iK and KN provided guidance and technical supports on IUE. WL and CT wrote the manuscript.

## Conflict of Interest Statement

The authors declare that the research was conducted in the absence of any commercial or financial relationships that could be construed as a potential conflict of interest.

## Abbreviations

BDNF, brain-derived neurotrophic factor; BPA, bisphenol A; BrdU, 5-bromodeoxyuridine; Cdk5, cyclin-dependent kinase 5; Cux2, cut-like homeobox 2; DISC1, disrupted in schizophrenia 1; E, embryonic day; GAPDH, glyceraldehyde-3-phosphate dehydrogenase; IUE, *in utero* electroporation; Kitl, kit ligand; Lhx2, LIM homeobox protein 2; MAP2, microtubule-associated protein 2; PBS, phosphate-buffered saline; PBST, PBS containing 0.05% Triton X-100; PFA, paraformaldehyde; PND, postnatal day; Slitrk1, SLIT and NTRK-like family, member 1; Tle3, transducin-like enhancer of split 3, homolog of Drosophila E; TrkB, neurotrophic tyrosine kinase, receptor, type 2.

## References

[1] Harley KG Gunier RB Kogut K Johnson C Bradman A Calafat AM Prenatal and early childhood bisphenol A concentrations and behavior in school-aged children. Environ Res (2013) 126:43–50. 10.1016/j.envres.2013.06.004 23870093 PMC3805756

[2] Hong SB Hong YC Kim JW Park EJ Shin MS Kim BN Bisphenol A in relation to behavior and learning of school-age children. J Child Psychol Psychiatry (2013) 54:890–9. 10.1111/jcpp.12050 23445117

[3] Evans SF Kobrosly RW Barrett ES Thurston SW Calafat AM Weiss B Prenatal bisphenol A exposure and maternally reported behavior in boys and girls. Neurotoxicology (2014) 45:91–9. 10.1016/j.neuro.2014.10.003 25307304 PMC4362616

[4] Yang CW Chou WC Chen KH Cheng AL Mao IF Chao HR Visualized gene network reveals the novel target transcripts Sox2 and Pax6 of neuronal development in trans-placental exposure to bisphenol A. PLoS One (2014) 9:e100576. 10.1371/journal.pone.0100576 25051057 PMC4106758

[5] Elsworth JD Jentsch JD Vandevoort CA Roth RH Jr DE Leranth C. Prenatal exposure to bisphenol A impacts midbrain dopamine neurons and hippocampal spine synapses in non-human primates. Neurotoxicology (2013) 35:113–20. 10.1016/j.neuro.2013.01.001 23337607 PMC3660149

[6] Mathisen GH Yazdani M Rakkestad KE Aden PK Bodin J Samuelsen M Prenatal exposure to bisphenol A interferes with the development of cerebellar granule neurons in mice and chicken. Int J Dev Neurosci (2013) 31:762–9. 10.1016/j.ijdevneu.2013.09.009 24091367

[7] Sadowski RN Wise LM Park PY Schantz SL Juraska JM. Early exposure to bisphenol A alters neuron and glia number in the rat prefrontal cortex of adult males, but not females. Neuroscience (2014) 279:122–31. 10.1016/j.neuroscience.2014.08.038 25193849 PMC4197082

[8] Tiwari SK Agarwal S Chauhan LK Mishra VN Chaturvedi RK Bisphenol-A impairs myelination potential during development in the hippocampus of the rat brain. Mol Neurobiol (2015) 51:1395–416. 10.1007/s12035-014-8817-3 25084756

[9] Kimura E Matsuyoshi C Miyazaki W Benner S Hosokawa M Yokoyama K Prenatal exposure to bisphenol A impacts neuronal morphology in the hippocampal CA1 region in developing and aged mice. Arch Toxicol (2015). 10.1007/s00204-015-1485-x 25804199 PMC4754327

[10] Fahrion JK Komuro Y Li Y Ohno N Littner Y Raoult E Rescue of neuronal migration deficits in a mouse model of fetal Minamata disease by increasing neuronal Ca2+ spike frequency. Proc Natl Acad Sci U S A (2012) 109:5057–62. 10.1073/pnas.1120747109 22411806 PMC3323999

[11] Guo BQ Yan CH Cai SZ Yuan XB Shen XM. Low level prenatal exposure to methylmercury disrupts neuronal migration in the developing rat cerebral cortex. Toxicology (2013) 304:57–68. 10.1016/j.tox.2012.11.019 23220560

[12] Gospe SM Jr Zhou SS. Prenatal exposure to toluene results in abnormal neurogenesis and migration in rat somatosensory cortex. Pediatr Res (2000) 47:362–8. 10.1203/00006450-200003000-00013 10709736

[13] Tabata H Nakajima K. Efficient in utero gene transfer system to the developing mouse brain using electroporation: visualization of neuronal migration in the developing cortex. Neuroscience (2001) 103:865–72. 10.1016/S0306-4522(01)00016-1 11301197

[14] Tabata H Nakajima K. Labeling embryonic mouse central nervous system cells by in utero electroporation. Dev Growth Differ (2008) 50:507–11. 10.1111/j.1440-169X.2008.01043.x 18482404

[15] Tomita K Kubo K Ishii K Nakajima K. Disrupted-in-schizophrenia-1 (Disc1) is necessary for migration of the pyramidal neurons during mouse hippocampal development. Hum Mol Genet (2011) 20:2834–45. 10.1093/hmg/ddr194 21540240

[16] Tomasi D Volkow ND. Abnormal functional connectivity in children with attention-deficit/hyperactivity disorder. Biol Psychiatry (2012) 71:443–50. 10.1016/j.biopsych.2011.11.003 22153589 PMC3479644

[17] Maximo JO Cadena EJ Kana RK. The implications of brain connectivity in the neuropsychology of autism. Neuropsychol Rev (2014) 24:16–31. 10.1007/s11065-014-9250-0 24496901 PMC4059500

[18] Nakamura K Itoh K Yaoi T Fujiwara Y Sugimoto T Fushiki S. Murine neocortical histogenesis is perturbed by prenatal exposure to low doses of bisphenol A. J Neurosci Res (2006) 84:1197–205. 10.1002/jnr.21020 16902998

[19] Nakamura K Itoh K Sugimoto T Fushiki S. Prenatal exposure to bisphenol A affects adult murine neocortical structure. Neurosci Lett (2007) 420:100–5. 10.1016/j.neulet.2007.02.093 17532137

[20] Yeo M Berglund K Hanna M Guo JU Kittur J Torres MD Bisphenol A delays the perinatal chloride shift in cortical neurons by epigenetic effects on the Kcc2 promoter. Proc Natl Acad Sci U S A (2013) 110:4315–20. 10.1073/pnas.1300959110 23440186 PMC3600491

[21] Melnick R Lucier G Wolfe M Hall R Stancel G Prins G Summary of the national toxicology program’s report of the endocrine disruptors low-dose peer review. Environ Health Perspect (2002) 110:427–31. 10.1289/ehp.02110427 11940462 PMC1240807

[22] Welshons WV Thayer KA Judy BM Taylor JA Curran EM vom Saal FS. Large effects from small exposures. I. Mechanisms for endocrine-disrupting chemicals with estrogenic activity. Environ Health Perspect (2003) 111:994–1006. 10.1289/ehp.5494 12826473 PMC1241550

[23] Vandenberg LN Non-monotonic dose responses in studies of endocrine disrupting chemicals: bisphenol A as a case study. Dose Response (2014) 12:259–76. 10.2203/dose-response.13-020.Vandenberg 24910584 PMC4036398

[24] Medina DL Sciarretta C Calella AM Von Bohlen Und Halbach O Unsicker K Minichiello L. TrkB regulates neocortex formation through the Shc/PLCgamma-mediated control of neuronal migration. EMBO J (2004) 23:3803–14. 10.1038/sj.emboj.7600399 15372074 PMC522798

[25] Wolstenholme JT Edwards M Shetty SR Gatewood JD Taylor JA Rissman EF Gestational exposure to bisphenol A produces transgenerational changes in behaviors and gene expression. Endocrinology (2012) 153:3828–38. 10.1210/en.2012-1195 22707478 PMC3404345

